# Formation of a large rice body-containing cyst following total hip arthroplasty

**DOI:** 10.1186/1756-0500-5-294

**Published:** 2012-06-14

**Authors:** Paul S Issack

**Affiliations:** 1 Department of Orthopaedic Surgery, New York Downtown Hospital, New York, USA

**Keywords:** Cyst, Pseudotumor, Rice bodies, Total hip arthroplasty

## Abstract

**Background:**

There are several well-described causes of a painful mass following total hip arthroplasty including polyethylene and metal wear debris, infection, expanding hematoma, dislocation, and synovial cysts. In addition to causing pain, these lesions, when large enough, may cause neurologic and vascular compromise. Rapid growth of the mass may clinically and radiographically resemble a sarcoma. Here, we report a case of a large painful hip mass which developed after total hip arthroplasty. The well-circumscribed mass was overlying and extending into the hip joint containing thousands of highly organized fibrin-containing “rice bodies”. To our knowledge, this is the first report of a large, highly organized (rice-body-containing) cyst complicating total hip arthroplasty.

**Case presentation:**

A 55-year old Caucasian woman developed a large, slowly enlarging, painful hip mass 2 1/2 years after primary total hip arthroplasty. Clinically and radiographically, the lesion resembled a soft tissue sarcoma. Surgical removal identified a well-circumscribed mass extending into the hip joint containing thousands of highly organized fibrin-containing “rice bodies”.

**Conclusion:**

Identification and excision of this “pseudotumor” following hip arthroplasty is important for obtaining a definitive diagnosis, ruling out malignancy or infection and relieving any potential compression on surrounding neurovascular structures.

## Background

The differential diagnosis of a painful mass in the hip following total hip arthroplasty includes polyethylene and metal wear debris [1-5], infection [6], hematoma [7,8], dislocation [9], malignancy such as synovial sarcoma, and malignant fibrous histiocytoma [10-12], synovial cyst [2,4], and iliopsoas bursitis [13].

We report here a case of a 55-year old woman who developed a large painful hip mass 2 1/2 years after primary total hip arthroplasty. The mass slowly increased in size and in clinical and radiographic presentation, resembled a soft tissue sarcoma on imaging. Surgical exploration identified a well-circumscribed mass overlying and extending into the hip joint containing thousands of highly organized fibrin-containing “rice bodies”. To our knowledge, this is the first report of a large, highly organized (rice-body-containing) cyst complicating total hip arthroplasty.

## Case presentation

**Figure 1:**
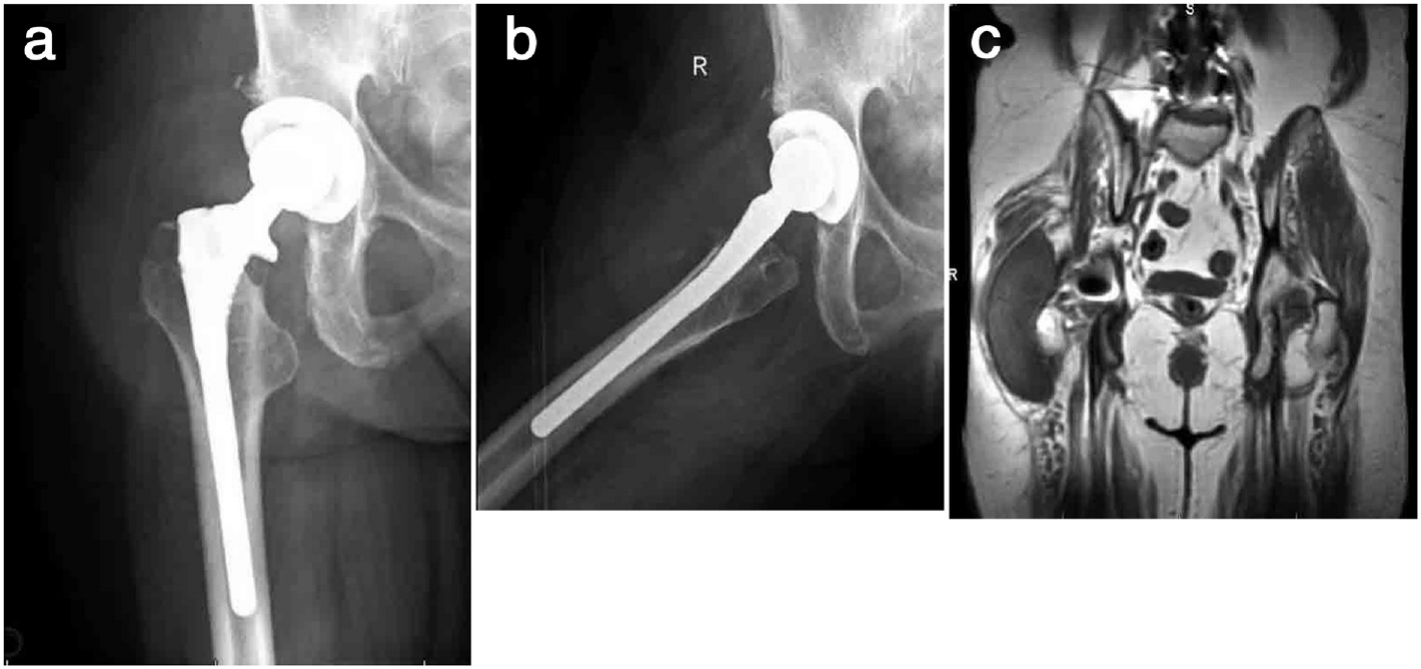
**a, Anteroposterior and b, lateral radiographs demonstrating an uncemented total hip arthroplasty. c**, Coronal T1-weighted MRI cut demonstrating the large well-circumscribed lesion overlying the right hip joint.

A 55-year old Caucasian female with a past medical history significant for hypertension and a lumbar laminectomy for spinal stenosis 5 years prior to the current presentation underwent a uncemented metal-on-polyethylene total hip arthroplasty 2 years prior to the current presentation using a Converge acetabular cup and APR (proximally hydroxyapatite porous-coated) stem (Zimmer, Warsaw, IN) for right hip osteoarthritis. She had no pain and functioned well in terms of ambulation and activities of daily living for the first two years. After two years, she began to notice enlargement of a mass over her right hip which began to make it increasingly difficult for her to sit or lie on the right side. She ambulated at this time with a Trendelenberg gait to the right side. Her posterolateral incision was well-healed and there was no warmth or erythema. She had a large palpable mass overlying the surgical incision. She had a painless range of motion of the hip and was neurovascularly intact distally with good distal pulses. Plain radiographs did not demonstrate radiolucencies at the implant-bone interface or eccentric polyethylene wear (Figure 1a,b). Stem position was unchanged from postoperative radiographs. Osteolysis was noted beneath the collar of the stem. A circular soft-tissue density was seen surrounding the hip joint (Figure 1a,b). Three- phase technetium bone scanning demonstrated increased blood pooling involving the soft tissue lateral to the prosthesis suggesting soft tissue injury, but no evidence of prosthetic loosening. There were no other areas of activity noted. Magnetic resonance imaging demonstrated a massive collection lateral to the hip joint with extension into the ilopsoas bursa. A thin capsule-type wall surrounded the mass (Figure 1c). The mass demonstrated intermediate signal on T1-weighted images and increased signal on T2-weighted images.

**Figure 2:**
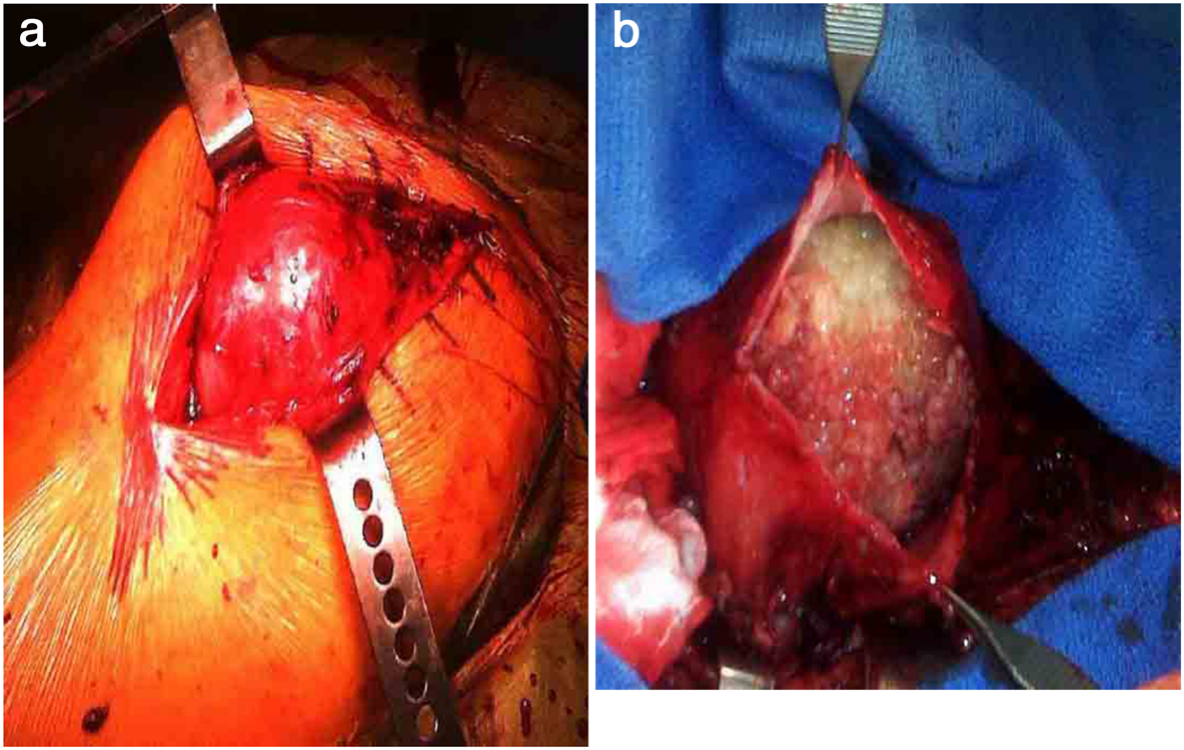
**a, Intraoperative photograph demonstrating the well-circumscribed cyst deep to the fascial layer and overlying the hip joint. b**, Contents of the cyst exposed.

Because of the patient’s persistent symptoms, the patient underwent surgical excision of the lesion through a posterior approach to the hip joint. After division of the tensor fascia lata and the gluteus maximus musculature, a large cystic structure protruded from the wound measuring 14 × 10 × 4 19 centimeters (Figure 2a). The cyst was noted to be compressing the sciatic nerve, although the patient did not demonstrate evidence of sciatic neuropathy. An attempt to aspirate the mass was unsuccessful. Therefore, a small incision was made in the capsular lining of the cyst to send a small quantity of the content for frozen section. Intraoperative frozen section revealed synovial hyperplasia with lymphocytic and neutrophilic infiltrate. Aggregates of organized fibrin (rice bodies) were noted. There was no evidence of infection. There was no evidence of malignancy, which would have required capsule closure, extraarticular resection of the hip joint and femoral prosthesis, and hip reconstruction. The cyst was then fully opened to reveal numerous granules of identical size and shape (Figure 2b). There was no gross necrotic tissue or hemorrhage. The contents of the cyst were emptied and the capsular lining was traced into the hip joint. The hip was dislocated and the capsular lining of the cyst was completely excised. There was no evidence of prosthetic loosening, polyethylene wear or metal debris. The wound was copiously irrigated and closed in layers over a drain. All cultures taken from the operating room, including aerobic, anaerobic, fungal, and acid fast bacilli, were negative for infection. Final histologic analysis confirmed the intraoperative frozen section report. Polyethylene or metallic wear debris were not identified. Following removal of the mass, the patient obtained immediate pain relief. An inpatient rheumatology workup failed to identify the presence of abnormal inflammatory markers such as rheumatoid factor or antinuclear antibody. On follow-up examination the patient was ambulating comfortably, and could sit and lie on her right side 2 months after excision of the cyst. These good results persisted to the latest follow-up 1 year after surgical excision.

## Discussion

There are several well-described causes of a painful mass following total hip arthroplasty. Many of the more common causes have characteristic physical exam, radiographic or serologic findings which make diagnosis relatively straightforward. For examples, following polyethylene and metal wear debris, hip arthroplasties have characteristic radiographic features including eccentric polyethylene wear, osteolysis [1-3,5], or a radiolucency outlining the effective joint space termed the “bubble sign” [14]. Infection can present with a mass if there is an infected hematoma or a large abscess [6,15]. In these situations, there may be prolonged wound drainage, radiographic changes, if the infection is chronic, or abnormal serology (elevated C-reactive protein and erythrocyte sedimentation rates) and positive cultures on joint aspiration [6,16]. An expanding hematoma may present with increasing size, correlating with a drop in the hematocrit and progressive sciatic nerve compression and palsy [7,8]. A periprosthetic dislocation would be readily identified on plain radiographs [9].

Rarer causes of painful hip masses following total hip arthroplasty have been described. Malignancies such as synovial sarcoma [10], osteosarcoma [11] and malignant fibrous histiocytoma [12] have been observed following total hip arthroplasty. Synovial cysts arising either from the hip joint capsule or from an expansion of the iliopsoas bursae following total hip arthroplasty are extremely rare, and there are only scattered case reports in the literature describing these [2-5,13]. In these cases, the leukocyte count, hemoglobin levels, hematocrit, erythrocyte sedimentation rate, and C-reactive protein levels were all within normal limits. In some cases, vascular compression by the cyst was what prompted exploration and surgical excision of the compressive mass [2,4,5]. In most of these cases, pathologic examination of these synovial cysts demonstrated fluid within the cyst with synovial cells on the inner surface of the cavity. Many of the specimens contained polyethylene debris suggesting that a hypersensitivity reaction to wear particles may be responsible for cyst formation [2,4,5]. In one case report, an allergic reaction to the cobalt-chromium molybdenum hip prosthesis was implicated in the development of a large firm soft tissue mass characterized by metallic debris and necrosis. Progessive sciatic nerve palsy resulting from compression by the mass prompted surgical excision [17].

The case reported here differs from those previously described in the literature in that the contents of the mass associated with the hip arthroplasty were highly organized. The mass contained numerous granules of fibrin or “rice-bodies” (Figure 2b). There was no evidence of polyethylene or metallic wear debris. There is one published report in the literature of a 83-year-old man who developed a large synovial cyst in his pelvis. The lesion was identified on computed tomography scanning and magnetic resonance imaging in the presacral area of the pelvic cavity. Surgical excision was performed. The cyst contained numerous fibrin-composed rice bodies. The cyst wall was composed of synovial tissue. The patient did not have a history of hip arthroplasty [18]. While rice bodies have been historically associated with rheumatoid arthritis and tuberculosis, this patient, similar to the one presented in this report, did not have evidence for either disease.

## Conclusion

While synovial cyst formation or iliopsoas bursitis complicating hip arthroplasties have been described in the literature, to our knowledge, this is the first report documenting the formation of a highly-organized fibrin-containing lesion following hip replacement. The growth and imaging characteristics of this lesion can resemble a soft tissue sarcoma. Thus, identification and excision of this “pseudotumor” is important for obtaining a definitive diagnosis and ruling out malignancy or infection. Furthermore, excision relieves the potential compressive effect of the mass on surrounding neurovascular structures including, as in this case, the sciatic nerve.

## Consent

Written informed consent was obtained from the patient for publication of this Case report and any accompanying images. A copy of the written consent is available for review by the Series Editor of this journal.

## Competing interests

The author declares that he has no competing interests.

## Author’s contributions

PI performed the surgery and drafted the manuscript. All authors read and approved the final manuscript.

## Author’s information

P.I. performed the surgical procedure, drafted and reviewed this case report.
